# Preventive Effects of Vanillic Acid Against Lung Inflammation and Oxidative Stress Induced by Dust Particles in Wistar Rats

**DOI:** 10.1111/jcmm.70573

**Published:** 2025-04-27

**Authors:** Seyedeh Samira Hosseinzadeh, Nazanin Balighi, Jafar Saeidi, Mohsen Azimi‐Nezhad, Mahnaz Mohtashami, Zahra Hojat Bonab, Mansoureh Dehghani, Mona Ariamanesh, Abolfazl Naimabadi, Ahmad Ghasemi, Amir Abbas Momtazi‐Borojeni

**Affiliations:** ^1^ Department of Biology, School of Basic Science Neyshabur Branch, Islamic Azad University Neyshabur Iran; ^2^ Department of Physiology, School of Basic Science, Neyshabur Branch Islamic Azad University Neyshabur Iran; ^3^ Healthy Ageing Research Centre Neyshabur University of Medical Sciences Neyshabur Iran; ^4^ Department of Microbiology, School of Basic Science, Bonab Branch Islamic Azad University Bonab Iran; ^5^ Department of Radiation Oncology Neyshabur University of Medical Sciences Neyshabur Iran; ^6^ Department of Pathology Neyshabur University of Medical Sciences Neyshabur Iran; ^7^ Department of Environmental Health Engineering, School of Public Health Neyshabur University of Medical Sciences Neyshabur Iran; ^8^ Department of Biochemistry, Nutrition and Food Sciences, School of Medicine Gonabad University of Medical Sciences Gonabad Iran; ^9^ Department of Medical Biotechnology, School of Medicine Neyshabur University of Medical Sciences Neyshabur Iran

**Keywords:** environmental pollution, heavy metal, inflammation, lung, oxidative stress, vanillic acid

## Abstract

To evaluate dose‐dependent cytotoxicity effects of indoor dust particles (DPs) collected from Neyshabur, Iran, in vitro on A545 cells and in vivo on lungs of healthy male Wistar rats, as well as the antioxidant effects of vanillic acid (VA) against DP inhalation. Heavy metal levels in DPs collected from high‐traffic (HT), medium‐traffic, low‐traffic or rural (LT) zones were measured, and their cytotoxicity effects were evaluated by MTT assay. In vivo evaluations were conducted after rats were exposed to DPs collected from HT or LT in the presence or absence of VA. Exposure to DPs increased the activity of serum superoxide dismutase; the serum level of malondialdehyde; and mRNA expression of TNFα, IL6, CXCL15 and CYP1A1 in the lung homogenate groups receiving HT and LT compared to the control group. DP effects in the groups receiving HT were higher than those of LT. Concomitant VA intake attenuated the adverse effects mediated by DPs in the HT and LT groups. DPs had adverse effects on the lungs of healthy rats, probably because of the accumulated oxidative stress agents. VA could ameliorate the effects of DPs and may be considered as a protective substance against the undesirable effects of DPs.

## 1 Introduction

Indoor dust particles (DPs) provide an ideal medium for the deposition of pollutants, such as heavy metals (HMs), pesticides and organic substances [1]. In this regard, the World Health Organization introduced DPs as toxic compounds for humans, due to their adverse effects on the respiratory, cardiovascular, digestive and nervous systems [2]. The undesired harmful impacts of indoor DPs on public health are gaining much attention because people spend substantial amounts of time in indoor environments. Previous studies detected a higher concentration of HMs in indoor DPs than in outdoor ones [3]. Spending more time and higher contaminant levels increase the exposure chance of indoor DPs as compared to outdoor ones [4]. HMs are dangerous pollutants in indoor dust due to some properties such as non‐degradability and high cytotoxicity [3]. Electrical devices, smoking tobacco and cigarettes, traffic sources, paints and types of cement used in buildings, lead‐acid batteries and outdoor dust are known major sources of HMs in indoor DPs [5, 6, 7]. Exposure to DPs can occur through the respiratory system, digestive system and skin as a result of living or working in places with high air pollution [3]. Congestion and traffic‐mediated pollution are typically the major contributors to air pollution in urban areas [8]. In this regard, previous reports have estimated a higher amount of organic and inorganic pollutants in HT zones than in low‐traffic or rural (LT) ones [9, 10]. Limited investigations have previously evaluated the cytotoxic effects of HMs derived from indoor dust in vitro [11, 12]. Previously, we revealed a high content of HM and cell cytotoxicity contributing to the DPs taken from outdoor and indoor environments [13]. Recently, the adverse effect of short‐term exposure to PM2.5 on lung tissue was reported by Li et al. [14]. In addition, it was reported that exposure to high concentrations of PM2.5 increased the content of collagen fibre and changed lung epithelial activity, the polarisation of immune cells and self‐degradative processes of cells in mice [15]. To the best of our knowledge, no documents about the potential cytotoxic effects of indoor DPs in vivo have been reported. In addition, there are still many gaps in our knowledge of how exactly indoor pollutants drive diseases. However, dysregulation of metabolic reactions, oxidative stress, proinflammatory conditions and tissue damage have been suggested to explain the adverse effects of DPs on the respiratory system [16, 17, 18, 19, 20].

Vanillic acid (VA), an acidic and aromatic substance, is biosynthesized through the shikimic acid pathway as well as oxidation of polyphenolic precursors in some plants [21]. Due to the chemical and structural properties [22] as well as wide usage as a food additive [21], the scientific interest in the beneficial effects of VA and its therapeutic potential has increased in recent decades. Phenolic acids such as VA have potent antioxidant properties, remove free radicals, mitigate oxidative stress and protect the cellular compound against oxidative radical damage [23] VA provides protective effects against oxidant agents, cancer development, melanogenesis and angiogenesis [24, 25]. It was revealed that VA decreases the malondialdehyde (MDA) level and has stimulatory effects on the activity of superoxide dismutase (SOD), catalase (CAT) and glutathione peroxidase (GPx) [26]. Previous studies have shown that VA attenuates the harmful effects of PM10 on electrocardiogram parameters, blood pressure and oxidative stress [27].

This study intends to investigate the toxic potential of indoor DPs in vivo and in vitro. The dose‐dependent cytotoxic effects of indoor DPs were investigated on the human alveolar A549 cells. Oxidative/anti‐oxidative analysis, proinflammatory changes and histopathological analysis were performed in rats. Moreover, the antioxidant effects of the VA administration against DPs' effects were evaluated in rats.

## 2 Materials and Methods

### 2.1 Sample Collecting

**FIGURE 1:**
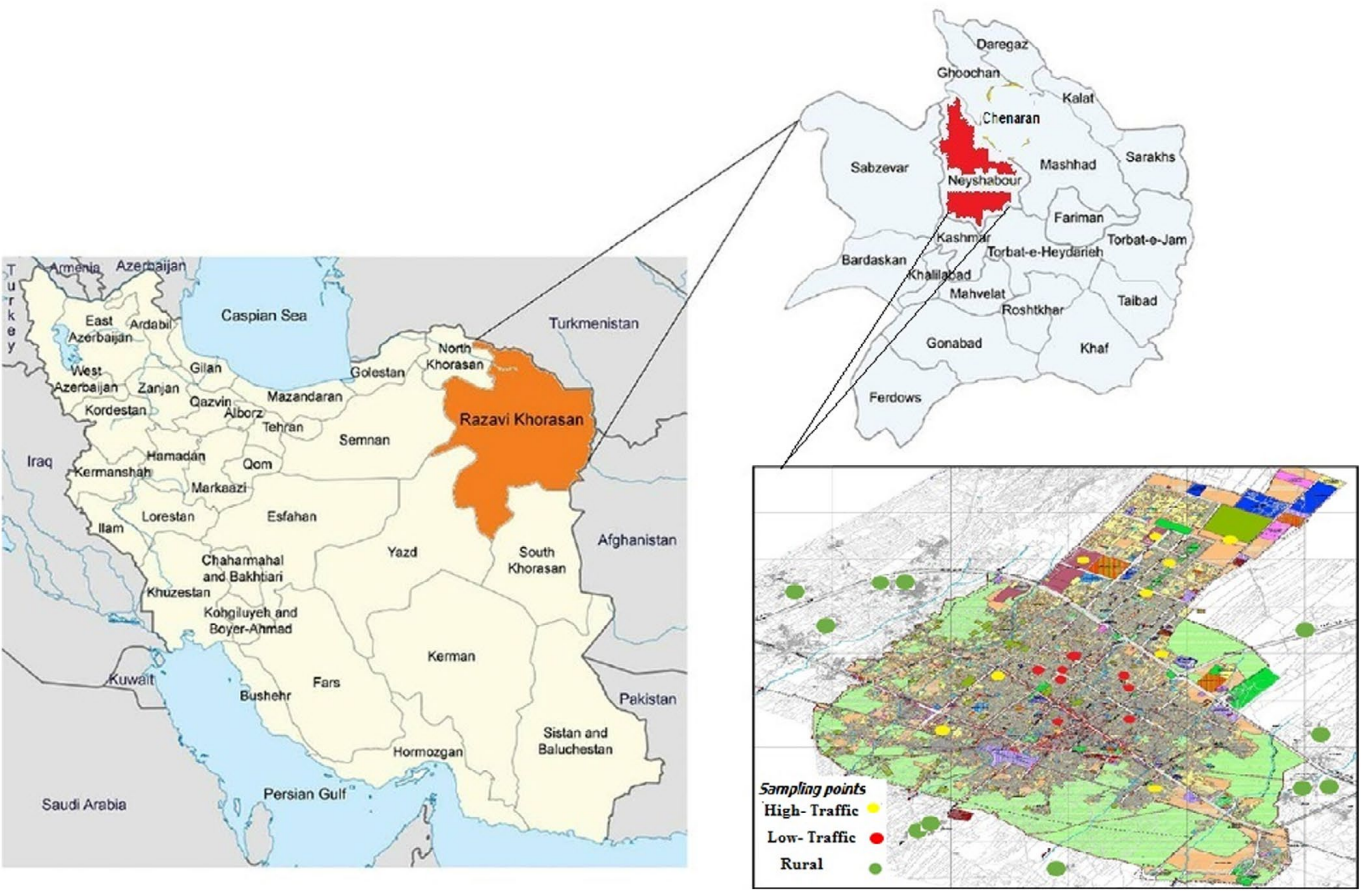
Location of the study area and sampling points [13].

The study was performed in Neyshabur city positioned in the range of 58° and 8′ to 59° and 20′ of longitude and 35° and 35′ to 36° and 52′ north latitude (Figure 1). Neyshabur is the second most populous city in the Khorasan Razavi province (Iran). The subject of HM pollution in Neyshabur has attracted substantial attention because of the presence of several industrial centres including the steel complex in its suburbs. In the current study, areas were separated into three traffic density categories: high‐traffic (HT), medium‐traffic (MT) and LT. The DPs from several houses in HT, MT and LT zones were collected from vacuum cleaners using a brush. To remove bulk particles, collected dust samples were sieved using a No. 40 mesh tool. The DP samples were kept in dust‐free zip lock bags and stored at room temperature until analysis.

### 2.2 Cell Culture

Lung carcinoma cell lines (A549) were purchased from the National Cell Bank of Iran (NCBI, Pasteur Institute of Iran). The cells were grown in RPMI‐1640 medium supplemented with 10% fetal bovine serum (FBS) along with penicillin G (100 U/mL) and streptomycin (100 μg/mL). The cell culture flask was maintained at a temperature of 37°C ± 0.5°C, 95% humidity and 5% CO_2_.

### 2.3 Cytotoxicity Assay

The 3‐[4,5‐dimethylthiazol‐2‐yl]‐2,5 diphenyl tetrazolium bromide (MTT) assay was used to evaluate the cytotoxic effects of DPs on A549 cells. A total of 5 × 10^4^ A549 cells were added to each well of a 96‐well plate, seeded in the RPMI medium with FBS (10%) and kept overnight. The cultured cells were exposed to concentrations of 50–200 μg/mL of the water‐soluble fraction of DPs from triple regions (LT, MT and HT). After a 72‐h incubation period, 20 μL MTT reagent 0.5 mg/mL was added to the mediums, and the cells were kept at 37°C for 4 h. The crystals of formazan in the cell culture medium were dissolved by adding 100 μL of DMSO (100%) solution. The optical density (OD) at 570 nm for the purple‐coloured solution of each well was measured using a microplate reader. The cell viability (%) was estimated from OD using the following formula: (mean OD of treated group/mean OD of non‐treated group) × 100.

### 2.4 Animals

Seventy male Wistar rats weighing 180–240 g were purchased from the Laboratory Animal Centre of Mashhad University of Medical Sciences. All Wistar rats were generally housed under a 12‐h light–dark cycle (25°C ± 2°C, 50% ± 10% humidity) with ad libitum access to food (Javaneh Khorasan Ltd., Iran) and water. All animal experiments were performed in compliance with the animal welfare guidelines reviewed and approved by the Institutional Ethics Committee and Research Advisory Committee of the Islamic Azad University of Neyshabur, Iran (IR, IAU.MSHD.REC 1401.002).

### 2.5 Experimental Design

The animals were treated according to a prior study performed by Dianat et al. [27] with minor modifications. The male rats were categorised into seven groups (*n* = 10): control group gavage with 1 mL normal saline (aqueous suspension) during 10 days, HT group (5 mg/kg DPs collected from HT zone, intratracheal instillation), LT (5 mg/kg DPs collected from LT zone, intratracheal instillation), HT/VA1 (5 mg/kg DPs collected from HT zone, intratracheal instillation; 10 mg/kg VA from Day 1 of DP installation), LT/VA1 (5 mg/kg DPs collected from LT zone, intratracheal instillation; 10 mg/kg VA from Day 1 DPs instillation), HT/VA3 (5 mg/kg DPs collected from HT zone, intratracheal instillation; 10 mg/kg VA3 after 3 days from DP instillation) and LT/VA3 (5 mg/kg DPs collected from LT zone, intratracheal instillation; 10 mg/kg VA after Day 3 from DPs instillation). Wistar rats received an aqueous suspension of VA (dissolved in normal saline) via a gavage needle for 10 days (animals were not anaesthetised). The DPs' intratracheal instillation was performed within 48 h as per the following protocol. The DPs were dissolved in sterile saline at certain concentrations and were constantly stirred on a magnetic stirrer for 20 min before use. Anaesthesia in rats was induced by the injection of ketamine (50 mg/kg) and xylazine (5 mg/kg). After anaesthesia, saline solution (0.1 mL)/DPs were instilled into the trachea via the intubation tube. After each run of intratracheal instillation, rats were mechanically ventilated for an additional 5 min. After 48 h, the animals were anaesthetised again, 1000 IU/kg of heparin sodium was administered intraperitoneally into the animal to inhibit blood coagulation, normal saline (0.1 mL)/DPs was instilled into the trachea, and the rats were ventilated for 5 min. Rats were humanely sacrificed through cervical dislocation after the final exposure day. After the dissection of the rats, the lungs were carefully excised from the chest and then weighed. A section of lung tissue was fixed for histological analysis, and another section was homogenised for mRNA isolation. A 5 mL sample of heart blood from each sacrificed animal was carefully taken. Clotted blood samples were subjected to centrifugation (1000 *g* for 10 min) to isolate serum sample and then were kept at −80°C for future analysis.

### 2.6 Histopathological Analysis

Each lung tissue sample was fixed with 10% neutral buffered formalin and routinely processed in paraffin. Specimens were sliced into 4–5 μm–thickness sections and then dewaxed and hydrated using a series of alcohol solutions of increasing concentration. The sections were placed on glass slides and allowed to adhere to the surface. The slides were then heated to ensure proper adhesion. Then, the paraffin wax was removed from the sections by soaking the slides in xylene or a similar clearing agent. The deparaffinised sections were rehydrated by passing them through a series of alcohol solutions of decreasing concentration. Staining of sections was performed by placing these in H&E solution (Sigma‐Aldrich, USA), haematoxylin for 7 min and eosin staining for 3 min at room temperature. The histopathological analysis of lung samples was established independently by two expert oncologists and pathologists based on microscopic examination and related criteria.

### 2.7 Oxidative/Anti‐Oxidative Analysis

Determination of oxidant/antioxidant analysis was performed by MDA and SOD analysis. SOD activity was determined according to the procedure of Fridovich et al. [28]. The MDA level in serum samples was measured to assess the degree of lipid peroxidation according to the procedure described by Ohkawa et al. [29].

### 2.8 Real‐Time PCR

The alterations in mRNA levels of tumour necrosis factor‐alpha (TNF‐α), chemokine ligand 15 (CXCL15), interleukin 6 (IL6), cytochrome p450 1A1 (CYP1A1) in lung tissues were determined by quantitative real‐time PCR. For this, lung tissue samples (30 mg) were homogenised in TRIzol reagent (Invitrogen, USA) and total RNA from each sample was extracted. Two micrograms of total RNA was used for cDNA synthesis using a Takara Shuzo cDNA synthesis kit (Otsu, Japan). Real‐time PCR with cDNA of each sample was performed on an ABI7500 sequence detection system (Applied Biosystems) using the SYBR green method (Amplicon, Denmark). PCR was run with the following PCR cycling program: an initial denaturation step at 95°C (15 min) followed by 40 cycles of 95°C for denaturation (30 s), 59°C for annealing (5 s) and 72°C for elongation (5 s). To check primer specificity, melting curves were produced at the end of each run. The relative mRNA level of the mentioned genes was normalised relative to GAPDH as the reference control gene. The fold change in target genes was calculated by the $2^{-\Delta \Delta C_{t}}$ method based on the *C*_t_ value. The sequences of primers for RT‐PCR analysis are presented in Table 1.

**TABLE 1:** Primers sequences used for RT‐qPCR.

Gene	Forward (5′–3′)	Reverse (5′–3′)
CXCL15	GGTGATATTCGAGACCATTTACTG	GCCAACAGTAGCCTTCACCCAT
TNFα	GGTGCCTATGTCTCAGCCTCTT	GCCATAGAACTGATGAGAGGGAG
CYP1A1	CATCACAGACAGCCTCATTGAGC	CTCCACGAGATAGCAGTTGTGAC
IL6	TACCACTTCACAAGTCGGAGGC	CTGCAAGTGCATCATCGTTGTTC
GAPDH	CATCACTGCCACCCAGAAGACTG	ATGCCAGTGAGCTTCCCGTTCAG

### 2.9 Statistical Analysis

Results were reported as mean ± standard deviation (SD) of three biological replicates. The statistical analysis was done using the SPSS software (18.0). A comparison of means in two groups was done using the *t*‐test. Comparison of means among multiple groups was done using the one‐way analysis of variance (ANOVA), followed by a Tukey post hoc test. *p* value < 0.05 was considered statistically significant.

## 3 Results

### 3.1 Dose‐Dependent Cytotoxicity Effects of Indoor DPs on A545 Cell Line

**FIGURE 2:**
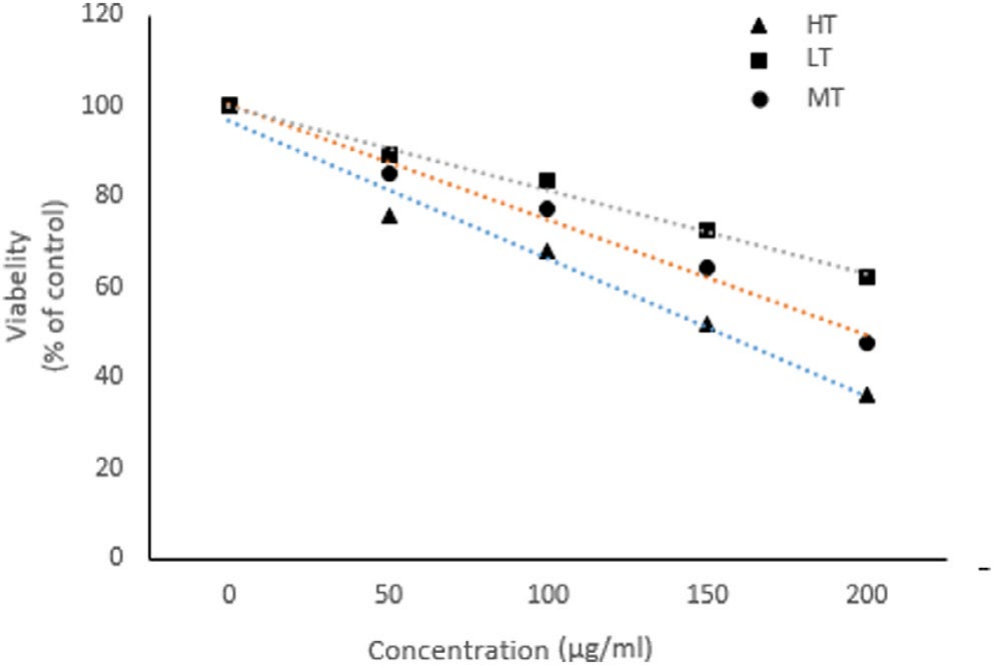
Dose‐dependent effects of indoor DPs on viability of lung cell line. A549 cells were cultured in a medium containing FBS (10%) overnight. Cultured cells were treated with various concentrations (0, 50, 100, 150 and 200 μg/mL) of low‐traffic or rural, medium‐traffic and high‐traffic samples for 48 h. The cell viability (%) in the treated cells was analysed using the MTT assay compared to untreated control cells (100%). The experiment was performed in triplicate, and values presented as mean ± SD of repeats.

The effect of treatment to different doses of indoor DPs from HT, MT and LT zones on the viability of A545 cells was measured using MTT. The results show a dose‐dependent decrease in the percentage of A545 cell viability following exposure to DPs collected from different zones, relative to the non‐treated control cells (Figure 2). The suppressive effects of indoor DPs on cell viability started at 50 mg/mL (% cell viability in HT: 75.62 ± 5; MT: 85.18 ± 10.3; LT: 88. 8 ± 3 in comparison to the control group, *p* < 0.05) and reached the maximum level at 200 mg/mL (% cell viability in HT: 36.41 ± 7.3; MT: 47.38 ± 1.2; LT: 61.94 ± 5.2 in comparison to the control group, *p* < 0.01). In addition, the inhibitory effects of DPs from the HT area were much greater than those of DPs collected from the MT and LT areas (*p* < 0.01).

### 3.2 Histopathological Response of Rats Receiving Indoor DPs

**FIGURE 3:**
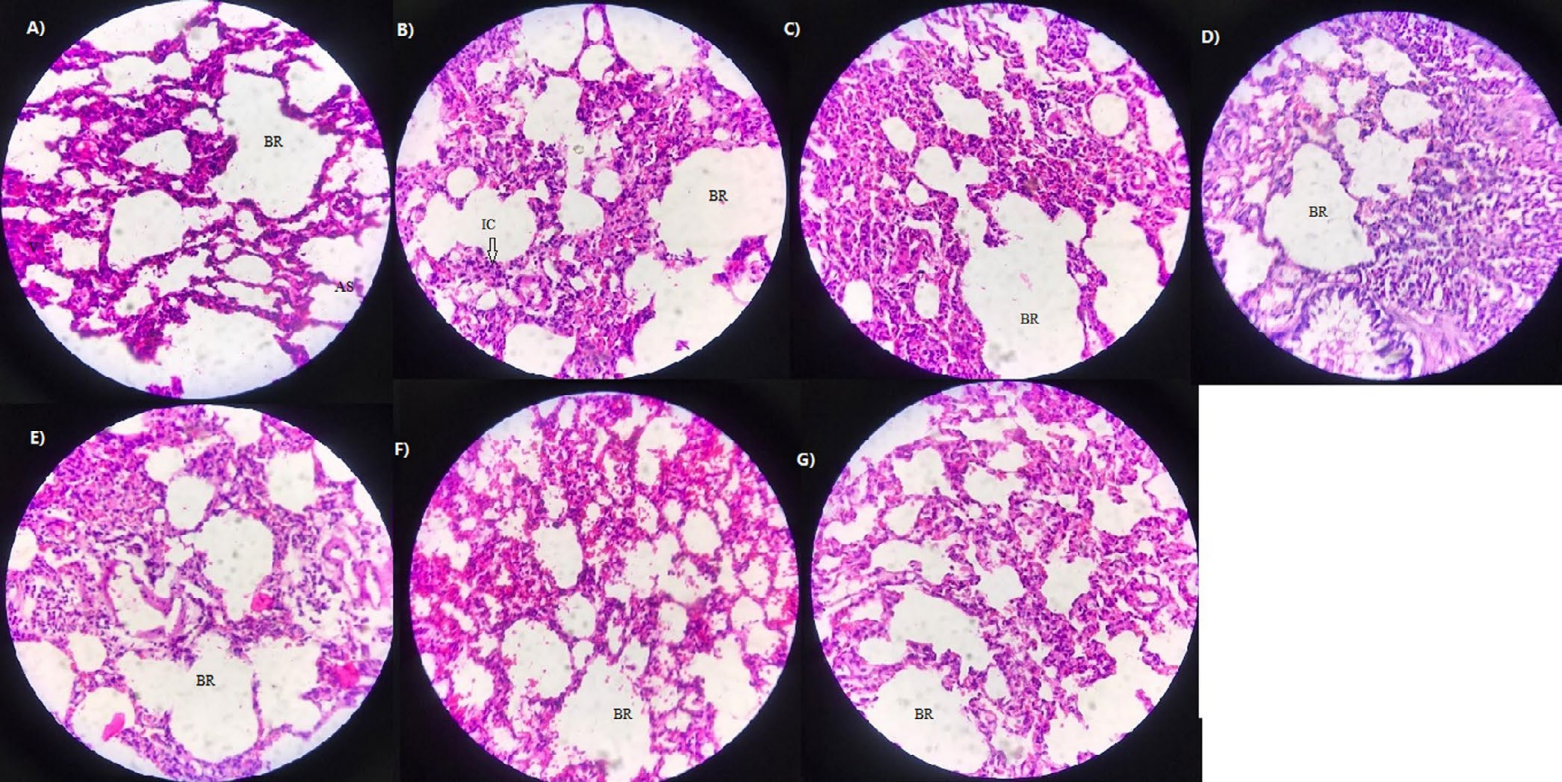
Pathological image of lung tissue samples from experimental groups by haematoxylin and eosin staining. (A) Control group demonstrating normal structure of lung tissue including vessels (V), bronchioles (BR) alveolar septa (AS). Different levels of thickened alveolar septa and infiltration of inflammatory cells (IC) into the alveoli were seen in the HT group, (C) HT/V1 group, (D) HT/V3 group, (E) LT group, (F) LT/V1 group, (G) LT/V3 group (original magnification ×400).

A summary of lung histopathological findings after DP exposure is demonstrated in Table 2. After DP exposure, there was mild to severe inflammation in the HT and HT/VA1 groups, whereas in the control groups, that is HT/VA3, LT, LT/VA1 and LT/VA3, mild‐to‐moderate inflammation was seen. There was also severe bronchiolitis and intraparenchymal haemorrhage in the HT group. The administration of VA after 3 days decreased bronchiolitis and intraparenchymal haemorrhage in the HT/V3 group. In addition, the proliferation in alveolar cells (pneumocytes type I) was seen in the HT and HT/VA1 groups, whereas treatment with VA inhibited cell proliferation in the HT/VA3 group. The results indicated that exposure to DPs collected from the HT zone induced severe adverse effects in the lung tissue, whereas VA showed a protective effect against DP activity in the lung tissue. The cross section of rat lung tissues stained with H&E (in different groups) is displayed in Figure 3.

**TABLE 2:** Lung histopathological findings of seven studied groups after treatment duration.

Group name	Severity of inflammation	Type of inflammatory cells	Bronchiolitis	Proliferation in alveolar	Intraparenchymal haemorrhage
Con (*n* = 4)	Mild to moderate	Lymphocyte Neutrophil	Mild	ND	Mild
HT (*n* = 4)	Moderate to severe	Lymphocyte Neutrophil	Severe	Was seen	Severe
HT/VA1 (*n* = 4)	Moderate to severe	Lymphocyte Neutrophil Eosinophil	Moderate	Was seen	Moderate
HT/VA3 (*n* = 4)	Mild to moderate	Lymphocyte Neutrophil	Mild	ND	Mild
LT (*n* = 5)	Mild to moderate	Lymphocyte Neutrophil	Mild	ND	Mild
LT/VA1 (*n* = 4)	Mild to moderate	Lymphocyte Neutrophil	Mild	ND	Mild
LT/VA3 (*n* = 5)	Mild to moderate	Lymphocyte Neutrophil	Mild	ND	Mild

### 3.3 Antioxidant Response of Rats Receiving Indoor DPs

**FIGURE 4:**
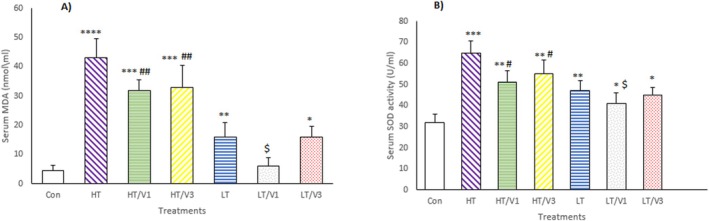
Oxidative stress markers analysis MDA level (A) and SOD activity (B) in the serum samples from different groups. *p < 0.05 versus control, **p < 0.01 versus control, ***p < 0.001 versus control, ****p < 0.0001 versus control; ^#^ *p* < 0.05 versus HT, ^##^ *p* < 0.01 versus HP; ^$^ *p* < 0.05 versus LT.

The oxidative potential of indoor DPs and antioxidant effects of VA were determined in rat groups HT, HT/VA1, HT/VA3, e LT, LT/VA1 and LT/VA3. Antioxidant response in the exposed rats was analysed by measuring the MDA level and SOD activity in serum. As shown in Figure 4, increased levels of MDA were detected in the HT (44 ± 6.5 nmol/mL; *p* < 0.0001) and LT (12.3 ± 3.5 nmol/mL *p* < 0.01) groups in comparison to the control group (6.3 ± 1.7 nmol/mL). In addition, the SOD activity was higher in the HT (70.3 ± 5.5 U/mL; *p* < 0.001) and LT (41.3 ± 3.5 U/mL; *p* < 0.01) groups than that of the control group (32 ± 4 U/mL). The effect on SOD activity and MDA level in the HT group was markedly higher relative to the LT group (*p* < 0.001). In the HT/VA1, HT/VA3 and LT/VA1 groups, the mean serum concentration of MDA and activity of SOD were lower than those of HT or LT groups. However, no significant differences were seen in the MDA serum concentration and SOD activity in the LT/VA3 relative to the LT group. These results demonstrated that being exposed to indoor DPs induces oxidative response, whereas receiving an antioxidant agent such as VA markedly suppresses it.

### 3.4 Inflammatory Response of Rats Receiving Indoor DPs

**FIGURE 5:**
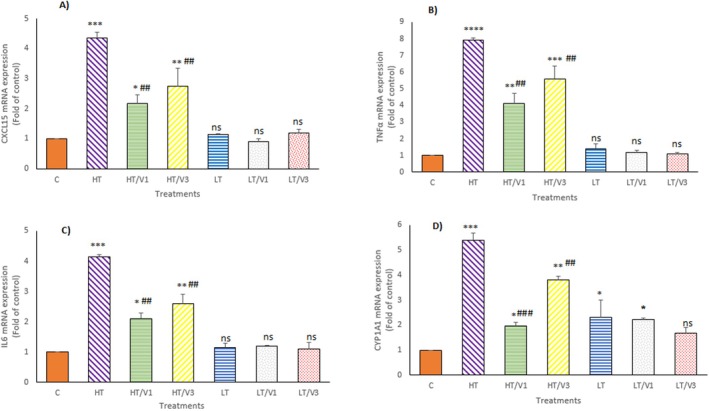
mRNA expression analysis of inflammatory cytokines CXCL15 (A), TNFα (B), IL‐6 (C) and CYP1A1 (D) in the lung tissue samples from different groups. Values are expressed as means ± SD. Significant differences are represented as follows: **p < 0.01 versus control, ****p < 0.0001 versus control; ^##^ *p* < 0.01 versus HT, ^###^ *p* < 0.001 versus HT, ns: not significant.

The indoor DP‐mediated inflammatory effects were determined by mRNA gene expression analysis of TNFα, IL6, CXCL15 and CYP1A1 genes in tissue samples of rats receiving indoor DPs. In the HT group, the mRNA gene expression of CXCL15 (*p* < 0.001), TNFα (*p* < 0.0001), IL6 (*p* < 0.001) and CYP1A1 (*p* < 0.001) was markedly enhanced relative to the untreated groups (Figure 5). In the HT/V1 and HT/V3 groups, the TNFα, IL6, CXCL15 and CYP1A1gene expression was significantly reduced relative to the HT group. Moreover, the mRNA level of CYP1A1 (*p* < 0.05) in the LT group was markedly greater than in the untreated group. No significant changes were observed in the expression of TNFα, IL6 and CXCL15 in response to LT exposure.

## 4 Discussion

There is evidence that indoor DPs have harmful effects on the health of urban residents [5]. It is well known that indoor DPs are composed of various HMs which are hazardous substances for the health of human subjects [5, 6]. Recently, the focus has shifted towards the HM pollution of Neyshabur, a city in Northeastern Iran. This city is located in the proximity of a steel complex and has an industrial nature, which, in turn, has diminished the quality of the environment in this region. In our previous investigations, we reported a comprehensive result from HMs in DPs collected from indoor and outdoor of HT, MT and LT areas. The results of HMs content in indoor DPs according to the traffic status were indicated. The highest concentration of HMs (μg/g) in indoor DPs was detected for Zn. The indoor DPs from the HT zone displayed higher content of Zn^+2^ (HT: 982.3 ± 12.6; MT: 726.3 ± 254; LT: 258.3 ± 106.3; *p* < 0.001) and Cu^+2^ (HT: 142.1 ± 17.4; MT: 137.1 ± 46.4; LT: 64.7 ± 29.2; *p* < 0.001) relative to MT and LT zones. The concentration of Ni^+2^ was higher in DPs from the LT zone than in HT and MT zones (HT: 94.1 ± 60; MT: 76.9 ± 32.4; LT: 154.9 ± 9.8; *p* < 0.01). The Pb^+2^ content of HT and MT DPs was higher than in the LT zone, whereas the Cr^+3^ level in DPs from HT and LT was greater than MT zone. The sum of measured HMs in HT DPs (1347.2) was markedly more than MT (1063.9) and LT (624.8) [13, 30]. In addition, cytotoxic effects of collected DPs from Neyshabur City on normal and cancerous cell lines were reported [31]. To our best knowledge to date, there is no data on the harmful effect of indoor DPs on histopathological response, inflammation and oxidative stress of lung tissue, as well as the protective effect of VA against the effects of DPs. Here, we analysed the effects of indoor DPs collected from Neyshabur on MDA levels, SOD activity and mRNA level of CXCL15, IL6, CYP1A1, CYP1B1 and TNF‐α genes in the lung tissue of seven rat groups and evaluated the protective effects of VA against these DPs. In addition, the dose‐dependent cytotoxic effects of indoor DPs from HT, MT and LT zones on A545 lung cells were evaluated.

MTT results showed that DPs from HT zones have higher cytotoxic effects on cell viability than those from MT and LT zones. This result can be attributed to a higher content of HMs in DPs from HT relative to MT and LT areas [32, 33]. The possible description regarding cytotoxic effects of HMs was reported by some researchers. Binding to DNA and RNA molecules, destroying the biomolecules through free radicals production and attenuating the antioxidant defence by glutathione molecule as a strong antioxidant are proposed mechanisms of HMs‐mediated cell death [12, 34, 35]. In agreement with our findings, dose‐dependent cytotoxicity effects of DPs were previously reported by others [20, 36, 37, 38].

The histopathological assessment showed severe inflammation, accompanied by bronchiolitis, proliferation in alveolar and intraparenchymal haemorrhage in animals exposed to indoor DPs from the HT area. Inhalation of indoor DPs leads to the suspension of particulates in the airways of animals, markedly dysregulating immune system responses and leading to inflammation in the lungs [39]. In this regard, Lei et al. also reported that Asian DP particles promote lung inflammation and injury in pulmonary hypertensive rats [40]. In addition, Pinho et al. demonstrated that intratracheal instillation of DPs in rats led to a potentiation of the inflammatory response and markedly increased oxidative damage in the lungs [41]. Moreover, subacute exposure of rats to lunar DP stimulant induced lung inflammation and oxidative stress damage by nitric oxide synthase and nicotinamide adenine dinucleotide phosphate oxidase overexpression [42].

Inhalation of environmental pollutants might lead to an increase in inflammatory cytokines in lung tissue [43, 44, 45, 46, 47]. A previous study reported that treatment with DPs increases the proinflammatory cytokines such as TNFα [18, 19, 48] and IL‐6 in lung tissue [18, 40, 49]. In line with the results from previous studies, we found that mRNA expression of proinflammatory cytokines such as TNF‐α and IL‐6 in the lung tissue of rats following exposure to indoor DPs was increased. Our findings also demonstrated that mRNA expression of the murine homologue of IL‐8, CXCL15, in rats after receiving indoor DPs was increased. Overexpression of IL‐6 and TNF‐α after exposure to indoor DPs may lead to the acute phase response, which is promoted by an IL8 and IL‐6 upregulation in the next round of cytokine release [39, 50]. Increased levels of DP‐mediated lung inflammation may cause coronary and carotid artery diseases [39, 51, 52]. In addition, previous studies have indicated that IL‐8, IL‐6 and TNF‐α are cytotoxic mediators of the link between inflammation and cancer [17]. Similar to cytotoxicity and pathophysiological results, increased inflammatory markers were higher in response to exposure to DP samples from HT areas than in LT areas. These results confirm previous studies regarding the role of traffic on environmental pollution and related side effects [7, 53].

DPs penetrate the airway, reach and deposit in the alveoli, and impair the respiratory system through reactive oxygen or nitrogen species (ROS, RNS) production and oxidative stress induction [54]. Redox active metals (Fe^3+^, Cu^2+^, Cr^3+^ Co^+3^ and other metals) are the most important sources generating ROS in response to indoor DPs exposure in live cells [17, 54]. In addition, direct evidence regarding the role of Cd, As, Ni, Cr and Fe in ROS production was proved experimentally [55, 56, 57, 58]. ROS or RNS induce DNA oxidative damage, lipid peroxidation and oxidative damage of vital proteins or enzymes [17]. However, antioxidant defences such as SOD enzymes protect cells against ROS by intercepting, scavenging and neutralising free radicals [17, 59]. The findings of the current investigation demonstrate that treatment with indoor DPs increased the serum level of MDA, a lipid peroxidation marker and the activity of SOD, an antioxidant enzyme. Consistent findings have demonstrated an increase in MDA levels and SOD in Wistar rats receiving PM10 [51, 60, 61]. The protective effects of VA against oxidative stress [24] and inflammation [62, 63] were previously reported in human cells. In addition, VA derivatives have shown analgesic and anti‐inflammatory activity [64]. Extensive studies have focused on VA, an acid derivative of vanillin, because of its anti‐hyperglycemic activity [65], anti‐oxidative [66, 67, 68], anti‐autoimmune nephritis [69], anti‐inflammatory [70] anti‐cancer [25] and antimicrobial [71] properties. It was demonstrated that VA pretreatment significantly improved the effects of PM10 on MDA levels, SOD activity, hemodynamic parameters and lipid peroxidation in rats [16, 70, 72]. In agreement with previous findings, our results have also shown that VA attenuates inflammation and oxidative stress mediated by indoor DPs [68].

## 5 Conclusions

In the previous investigation, we reported a high concentration of HMs and cell cytotoxicity in the indoor and outdoor DPs environments collected from LT, MT and HT areas [13]. Current results provide further analysis regarding the harmful effects of these DPs in the lungs of healthy rats. In this research, it was demonstrated that DPs develop a low‐grade inflammation probably due to a high oxidative stress index and low antioxidant agents in healthy rats. However, the administration of the anti‐oxidative compound VA reversed the harmful effects of DPs. Together with previous experiments, our findings reveal that exposure to indoor DPs may trigger or accelerate the development of inflammatory diseases in the lungs, and VA treatment can protect against adverse effects of DPs.

## Author Contributions

**Seyedeh Samira Hosseinzadeh:** validation (equal), writing – original draft (equal). **Nazanin Balighi:** investigation (equal), validation (equal). **Jafar Saeidi:** investigation (equal), validation (equal). **Mohsen Azimi‐Nezhad:** methodology (equal), validation (equal). **Mahnaz Mohtashami:** formal analysis (equal), validation (equal). **Zahra Hojat Bonab:** validation (equal), writing – review and editing (equal). **Mansoureh Dehghani:** data curation (equal), validation (equal). **Mona Ariamanesh:** formal analysis (equal), validation (equal). **Abolfazl Naimabadi:** investigation (equal), validation (equal). **Ahmad Ghasemi:** conceptualization (equal), funding acquisition (equal), project administration (equal), validation (equal). **Amir Abbas Momtazi‐Borojeni:** conceptualization (equal), methodology (equal), validation (equal).

## Ethics Statement

The animal study was reviewed and approved by the Animal Ethics Committee of Islamic Azad University of Neyshabur under Ethics number IR, IAU.MSHD.REC 1401.002.

## Consent

All authors agreed to publish current work in the Environmental Science and Pollution Research journal.

## Conflicts of Interest

The authors declare no conflicts of interest.

## Data Availability

All data analysed during the current study are available from the corresponding author upon reasonable request.
